# An Updated Systematic Review and Meta‐Synthesis of Service User's Experiences of Cognitive Behavioural Therapy for Psychosis (CBTp)

**DOI:** 10.1002/cpp.70356

**Published:** 2026-11-05

**Authors:** Peter Panayi, Katherine Berry, Aabhya Parnami, Alison Branitsky, Dung Jidong

**Affiliations:** ^1^ Division of Psychology and Mental Health University of Manchester Manchester UK; ^2^ Complex Trauma and Resilience Research Unit Greater Manchester Mental Health NHS Foundation Trust Manchester UK; ^3^ Psychosis Research Unit Greater Manchester Mental Health NHS Foundation Trust Manchester UK; ^4^ Faculty of Arts, Social and Management Sciences Coal City University Enugu Nigeria; ^5^ Mersey Care NHS Foundation Trust Liverpool UK

**Keywords:** CBT, hallucinations, paranoia, qualitative, schizophrenia, thematic analysis

## Abstract

Over the past 20 years, cognitive behavioural therapy for psychosis (CBTp) has seen worldwide implementation in mental health services. Systematic reviews and meta‐syntheses have identified common and specific therapeutic factors in CBTp, yet its efficacy remains modest. Since its global implementation, a myriad of qualitative studies have explored service users' experiences of CBTp but have yet to be comprehensively integrated in one meta‐synthesis to identify paths forward. A thematic meta‐synthesis of qualitative studies examining service users' experiences of CBTp was conducted according to a pre‐registered protocol. Systematic searches of CINAHL, Embase, PsycInfo, PubMed and MEDLINE databases were conducted in May 2026. Independent raters screened results for eligible papers and assessed their quality using the Critical Appraisal Skills Programme. Results are reported in accordance with PRISMA guidelines. Twenty‐four studies were included in the analysis, with a pooled sample of 334 participants. Three interconnected themes were generated: *active collaboration empowers me; promoting safety makes me feel more trusting; developing shared understandings helps me accept or challenge upsetting experiences*. They show that CBTp techniques promote acceptance and new meanings if sensitively and collaboratively used and collaboration in CBTp facilitates therapeutic outcomes via empowerment. Both relationships occur only when participants feel safe. In the largest review to date, these themes indicate the pivotal importance of collaboratively building safety and acceptance in facilitating therapeutic gains in CBTp. The development of scalable talking therapies should not compromise idiosyncrasy and digitisation of CBTp should carefully avoid compromising the therapeutic relationship for the sake of challenging appraisals.

**Key Practitioner Message**

- CBTp practitioners should avoid sacrificing idiosyncrasies for the sake of adherence to manualised protocols.
- Promoting safety should be prioritised to facilitate therapeutic gains.
- Accepting distressing experiences can be as beneficial as challenging appraisals.
- Digitisation of CBTp should not compromise a collaborative therapeutic alliance.

## 1 Introduction

Schizophrenia spectrum disorders (broadly referred to as ‘psychosis’) are associated with significant burden to the individual and society (Solmi et al. 2023). Several interventions have demonstrated efficacy in reducing the impact of psychosis, chiefly cognitive behavioural therapy for psychosis (CBTp) (World Health Organisation [WHO] 2023). CBTp aims to reduce distress, promote adaptive coping and support people to meet their goals (Fowler et al. 1995). It has seen worldwide implementation in mental health care following recommendation in several national guidelines, including but not limited to Europe, North America and Australia (Burgess‐Barr et al. 2023). But meta‐analyses show that CBTp effect sizes are modest and heterogeneous (Newman‐Taylor and Bentall 2024; Solmi et al. 2023). Identifying routes to maximise its efficacy is required, especially considering the resource investment required to deliver CBTp (Konings et al. 2025).

Cognitive models of psychosis on which CBTp is based imply that unhelpful appraisals of intrusive experiences (e.g., distressing beliefs; unshared sensations) precipitate and maintain distress and maladaptive coping (Garety et al. 2001). Yet, challenging appraisals in CBTp inconsistently predict therapeutic gains (Mehl et al. 2018; Morrison et al. 2012). Behavioural components of such models suggest that distressing appraisals incite safety behaviours and avoidance that counterintuitively maintain distress by preventing their disconfirmation (Morrison 2001). A foundation of CBTp practice is therefore collaborative empiricism: teaching people to be scientists by testing new ways of responding to distressing experiences (Morrison 2017). Fostering coping in this way temporally predicts reductions in suspiciousness but not other experiences, like upsetting voices (Schlier et al. 2020). From an interventionist‐causal perspective, the inconsistent efficacy of CBTp may therefore indicate an incomplete understanding of change mechanisms, requiring further investigation to sufficiently target said mechanisms, maximising its efficacy.

CBT has received criticism for its individualist perspective. For instance, some people experience disproportionate persecution or harm due to systemic bias and oppression (Pittman 2020; Wesely and Gaarder 2004). Challenging hypervigilance in CBTp may therefore be felt by recipients as dismissive or invalidating and unhelpful. Likewise, limits in human epistemic distance mean that some appraisals (e.g., the omnipotence of religious voices) are difficult to empirically test in a culturally‐congruent way (Cook 2018). These criticisms are most often experienced by people of global majority ethnicity, a group associated with considerably higher risk of attracting schizophrenia‐spectrum diagnoses than White people (Rathod et al. 2010; Qassem et al. 2014). Best practice guidance recommends therapists carefully negotiate which appraisals are re‐evaluated in CBTp, balancing identifying and modifying cognitive biases with validating disempowered experiences (Jagtap et al. 2024). Whether CBTp recipients have experienced and valued person‐centred practices of this kind remains unclear. It is important to establish because over‐adherence to individualist perspectives may contribute to aforementioned heterogeneity in CBTp outcomes.

Of course, the efficacy of all psychological interventions hinges on engagement (Lawlor et al. 2017). Aside from facilitating the application of change strategies, engagement allows for the development of a therapeutic alliance, considered instrumental to the effectiveness of psychological interventions, including CBTp (Bourke et al. 2021). Promoting engagement among people with psychosis specifically can be challenging, owing to historical and current experiences of enforced treatment and restrictive practices (Carr et al. 2018). These experiences have exclusionary consequences, including individual re‐traumatisation and group confirmation of distrust against mental health services, especially among minoritised ethnic groups (Payne‐Gill et al. 2021). This is not to mention the overwhelming rates of interpersonal victimisation and associated post‐traumatic stress among people with psychosis that may complicate the development of therapeutic relationships (Hardy et al. 2022).

Thus, improving CBTp efficacy may rely on the identification of change mechanisms as well as engagement strategies. Characterising recipient's experiences of CBTp may support this identification. For instance, the first meta‐synthesis of its kind identified two themes delineating common (e.g., power and self‐concept, acceptance) and specific factors (e.g., coping strategies and alternative explanations) involved in psychological interventions for psychosis (Berry and Hayward 2011). A subsequent meta‐synthesis of service users' experiences of individual CBTp identified additional themes, including the building blocks of a therapeutic alliance and challenges to applying CBTp (Wood et al. 2015). These findings had important implications for CBTp protocols, such as emphasising the importance of promoting engagement throughout (Morrison 2017). To implement this effectively, meta‐competencies in the training of CBTp therapists as genuine, collaborative and flexible are prioritised alongside technical competencies (Landa 2017).

Despite these implementations, the efficacy of CBTp remains modest, with up to a third of recipients reporting no recovery (Ludwig et al. 2026). Contemporary clinical trials aim to adjust CBTp to augment outcomes, targeting specific symptoms (e.g., post‐traumatic stress; Peters et al. 2026) or causal mechanisms (e.g., agoraphobia; Freeman et al. 2024). However, targeted interventions have demonstrated mixed efficacy, with some reporting no effect and others demonstrating improved outcomes on core psychopathology (Sitko et al. 2020). A wealth of qualitative studies on service user's experiences of CBTp have been published since its worldwide implementation, supporting refinement of the intervention for individuals for whom it has been ineffective. We therefore completed a systematic review and meta‐synthesis of this literature, centring service user perspectives to mitigate the re‐enactment of systemic oppression by the mental health system. With the aim of identifying routes to improve CBTp efficacy, perhaps via engagement and change mechanisms, we asked: What is the service user experience of CBTp?

## 2 Method

### 2.1 Design

The study design employed a systematic review and meta‐synthesis of qualitative studies of service users' experiences of CBTp, using Thomas and Harden's thematic synthesis framework (Thomas and Harden 2008).

### 2.2 Inclusion Criteria

From the first available record, studies published in peer‐reviewed academic journals from any country aimed at exploring service user's experiences of individualised CBTp were included in this review. CBTp was defined by NICE guideline CG178. Included studies were required to utilise widely‐accepted qualitative data collection methods (i.e., interviews, focus groups, direct observation and participatory action research) and describe their results in sufficient detail that allowed service user's accounts to be separated from others (e.g., therapists; support network). Studies were also required to involve participants meeting DSM‐5 or ICD‐10 diagnostic criteria for a schizophrenia‐spectrum disorder **and/or** currently or historically received support from an Early Intervention in Psychosis (EIP) service. Experiences of EIP service users were included since, in the UK, CBTp is most routinely practiced in such services. EIP best practice guidance advises against the use of potentially stigmatising labels (e.g., schizophrenia, schizoaffective disorder) among those experiencing first‐episode psychosis. Instead, ICD‐10 F28 (other non‐organic psychotic disorder) or F29 (unspecified psychotic disorder) categories are routinely applied.

### 2.3 Exclusion Criteria

Studies were excluded if they involved participants under age 14 (i.e., the age at which service users are accepted into EIP services in the UK) or who did not meet criteria for a DSM‐5 or ICD‐10 schizophrenia‐spectrum diagnosis and/or who are/have not accessed an EIP service. Studies on group, third‐wave and CBT‐informed interventions not recommended in UK NICE guidelines for the treatment of psychosis (e.g., cognitive analytic therapy; cognitive remediation) were also excluded. Publications that were not primary research (e.g., commentaries, protocols and systematic reviews) or studies for which it was difficult to extract qualitative data (e.g., mixed methods studies without clearly labelled data, or studies in settings where perceptions of service users around CBTp could not be clearly identified, such as summaries or aggregated data) were also excluded.

### 2.4 Search Strategy

This review was conducted in accordance with the Preferred Reporting Items for Systematic reviews and Meta‐Analyses (PRISMA (Page et al. 2021)) guidelines and according to a pre‐registered protocol on PROSPERO (ID CRD420251112606). Systematic searches of CINAHL, Embase, PsycInfo, PubMed and MEDLINE were conducted in August 2025 and updated in May 2026. Free‐text terms were used to search title, abstract and keyword fields and were grouped using the SPIDER framework (Cooke et al. 2012). Search terms and relevant Boolean operators are presented in Table 1. The search terms were based on the most similar historic review to facilitate comparability (Wood et al. 2015). Following librarian consultation, ‘evaluation’ terms were not included, since qualitative studies often conflate this construct with Design and Research type, risking over‐filtering and reduced sensitivity. Backward and forward citation searches of included studies and comparable historic reviews (Cox and Miller 2021; Pipkin, Hogg, and Armitage 2021; Wood et al. 2015) were also completed.

**TABLE 1:** Search terms according to corresponding SPIDER components and Boolean operators.

SPIDER tool^a^	Search terms
Sample	‘psychosis’ OR ‘schizophrenia’ OR ‘voices’ OR ‘delusions’ OR ‘hallucinations’ OR ‘schizoaffective’
Phenomenon of Interest	‘cognitive behaviour* therapy’ OR ‘CBT’ OR ‘CBTp’ OR ‘cognitive therapy’
Design	‘interview*’ OR ‘focus group*’
Research Type	‘qualitative’

^a^ S AND P of I AND [D OR R].

### 2.5 Study Selection and Data Extraction

Search results were entered into and assessed using Microsoft Excel. Following the identification and deletion of duplicate results, PP reviewed titles, abstracts and full texts sequentially against the inclusion criteria. To mitigate the risk of bias, a second reviewer (AP) independently screened all non‐duplicate results, achieving 98.5% agreement (Cohen's *κ* = 0.82; SE = 0.07), considered a strong level of agreement (McHugh 2012). Disagreements regarding the inclusion of articles were discussed and resolved with input from co‐authors DJ and KB. Once eligible papers were identified, results sections were extracted along with study characteristics (i.e., author, year of publication, study title, research design, setting, participant characteristics and analytical approach). To assess acceptability, service user perspectives were privileged, meaning data from clinicians, family members or laypeople were excluded.

### 2.6 Quality Assessment

Study quality was assessed using the Critical Appraisal Skills Programme (CASP 2024) framework, a 10‐item checklist designed to assess the quality of qualitative research studies. To stratify studies by methodological quality, checklist items were assigned a numerical value (Yes = 1; Cannot Tell = 0.5; No = 0), allowing the identification and exclusion of low‐quality studies from the synthesis to avoid compromising the findings (Long et al. 2020). Based on a maximum score of 10, studies were categorised into low (≤ 4), moderate (5–7) and high quality (8–10). PP and AP independently reviewed included studies against the CASP checklist, achieving 70.8% agreement, with disagreements resolved using consensus.

### 2.7 Analysis

Thomas and Harden's approach to meta‐synthesis was adopted (Thomas and Harden 2008). Results sections of included studies, including participant quotes and author summaries, were extracted and inductively coded line‐by‐line by PP to capture meaning. These codes were then compared across studies to identify similarities and differences and grouped to form descriptive themes reflecting key findings of the primary studies. PP and AP independently constructed descriptive themes in this way and compared results, triangulating these with input from DJ and KB to diversify perspectives on the analysis. Through iterative discussion, descriptive themes evolved from a longitudinal representation of participants' journeys through CBTp to four broader components explaining patterns across the dataset. Descriptive themes were themselves used to generate analytic meta‐themes reflecting the synthesist's interpretation, also iterated upon via discussion among the research team (Thomas and Harden 2008). For instance, descriptive sub‐themes, *Therapeutic Style*, *CBTp techniques* and *Outcomes* were taken to indicate the importance of *Sensitively applied CBTp techniques* and linked to *Thinking differently about myself and my future and Accepting my experiences*. Informed by grounded theory, a constant comparison approach was taken to developing the final thematic structure (Barnett‐Page and Thomas 2009). Newly generated analytic themes were iteratively refined through comparison with free codes, descriptive themes and superordinate themes, until the research questions were fully answered and the descriptive themes were explained (Thomas and Harden 2008). For instance, *Integrating technology* was relegated from theme to sub‐theme upon integration of other *Non‐specific factors* represented by additional codes.

### 2.8 Reflexivity and Positionality

The first author is a trainee clinical psychologist with 8 years of clinical and research experience in psychosis, including practice as a CBT therapist. Co‐authors DJ, KB and AB are also psychologists with considerable experience in clinical research and practice in psychosis, which may have influenced the interpretation of results in line with CBTp protocols and associated change strategies. PP and AB both have lived experience of mental health difficulties including a schizophrenia‐spectrum diagnostic label. Their lived experience influenced the decision to privilege participant experiences of CBTp over others (e.g., therapists; family/friends/carers). All authors are committed to the values underpinning ‘good’ CBT (including collaboration, inclusivity and autonomy), which may have guided the over‐attending to positive experiences in our findings and understanding negative experiences as circumstantial.

We adopted a constructionist position to the preparation of this work. We conceptualised each study to reflect their own reality, given their different philosophies that violated the positivist assumption they could represent a single, objective reality. Likewise, the generation of analytic themes according to Thomas and Harden's approach will be inexorably linked to the authors' positions outlined above, invalidating a positivist perspective that our themes would be replicated by every (or indeed any) other research team. Our constructionist position therefore allowed for the generation of higher‐order analytic themes, based on our and participants' experiences, that integrated conflicting perspectives.

## 3 Results

### 3.1 Study Characteristics

Twenty‐four studies were included in the synthesis, published between 2002 and 2024 (see PRISMA diagram in Figure 1). All studies used semi‐structured interviews aside from one, which used group game design (Slater and Painter 2016). Analytic frameworks included thematic analysis (*k* = 9), grounded theory (*k* = 5), content or systematic content and question analysis (*k* = 4), discourse analysis (*k* = 2), interpretive phenomenological analysis (*k* = 2) and framework analysis (*k* = 1).

**FIGURE 1:**
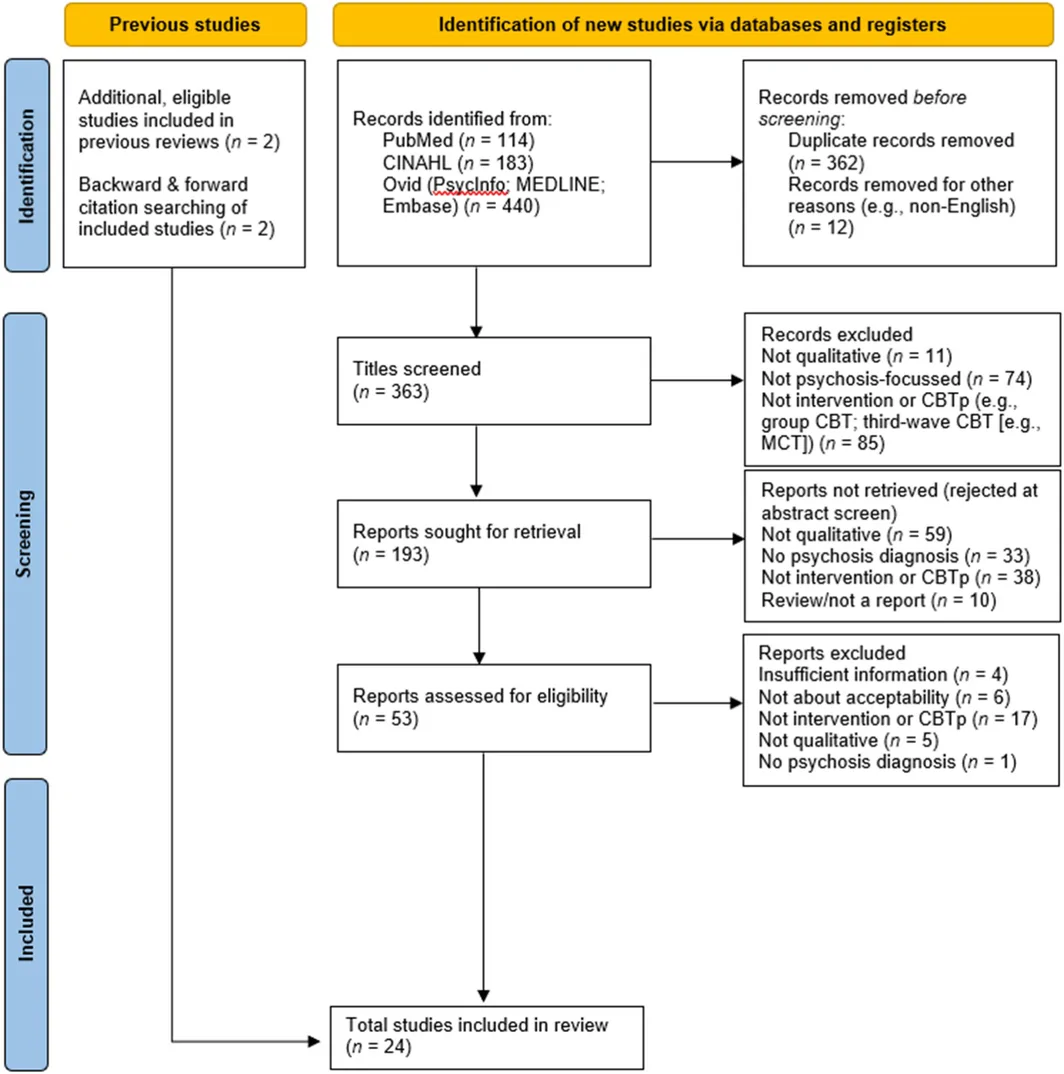
Flow chart of studies included in the review, adapted from Page et al. (2021).

There was a pooled sample of 334 participants with lived experience of psychosis and CBTp across the studies. Most studies were conducted in Western countries, including the UK (*k* = 16), Canada (*k* = 2), Europe (*k* = 2) and Australia (*k* = 1). The remaining studies were conducted in China (*k* = 1), Pakistan (*k* = 1) and Saudi Arabia (*k* = 1). These include *n* = 177 males and *n* = 148 females who took part, with sex not specified in one study (Health Quality Ontario 2018). Participants were primarily adults (*μ* $\bar{x}$ = 34.9 years; range 15–65), with mean age unreported in three studies (Gibbs et al. 2020; Health Quality Ontario 2018; Rathod et al. 2010). Detailed characteristics of included studies are listed in Table 2.

**TABLE 2:** Characteristics of included studies.

Study	Method	Sampling method	Data collection method	Participants	*N (*Male *n*)	Mean Age (Range)	Ethnicity
Aljuhani et al. (2024)	Framework analysis	Convenience	Semi‐structured interviews	Outpatient adults with a schizophrenia diagnosis and family members^a^	15 (10 male)	36.5 (22–65)	Unknown
Austin et al. (2021)	Mixed method/Thematic analysis	Convenience	Semi‐structured interviews	Adults with an ICD‐10 schizophrenia spectrum diagnosis	16 (4 male)	24.1 Range unknown	Unknown
Birchwood et al. (2018)	Thematic analysis	Random	Semi‐structured interviews	Adults with an ICD‐10 schizophrenia spectrum diagnosis	25 (15 male)	42.2 (18–67)	19 White British or other White background 6 Black African or Caribbean or Other
Dellazizzo et al. (2020)	Content analysis	Convenience	Therapist notes and open interviews	Adults with treatment‐resistant persecutory voices	10 (8 Male)	43.4 Range unknown	10 White
Dunn et al. (2002)	Grounded theory	Convenience	Semi‐structured interviews	Adults with lived experience of psychosis	10 (6 Male)	37.5 (31–52)	Unknown
Gibbs et al. (2020)	Grounded theory	Convenience	Semi‐structured interviews	Adults with lived experience of psychosis and their therapists^a^	11 (8 Male)	Mean unknown (21–65)	5 White British or other White background 3 Black African or Caribbean 3 Mixed heritage
Hall et al. (2023)	Grounded theory	Unknown	Semi‐structured interviews	Adults who hear voices	12 (8 Male)	50.3 (23–62)	11 White British 1 Asian/Asian British
Holgersen et al. (2023)	Thematic analysis	Convenience	Semi‐structured interviews	Teenagers with an ICD‐10 schizophrenia spectrum diagnosis	16 (4 Male)	16 (14–18)	Unknown
Health Technology Ontario (2018)	Grounded theory	Convenience	Semi‐structured interviews	Adults with lived experience of psychosis and caregivers^a^	7 (Male *n* unknown)	Unknown	Unknown
Kelly et al. (2012)	Discourse analysis	Convenience	Semi‐structured interviews	Adults with lived experience of psychosis and therapists^a^	4 (3 Male)	Mean unknown (26–45)	2 White British 2 Black British
Kilbride et al. (2013)	Interpretive Phenomenological Analysis	Convenience	Semi‐structured interviews	Adults with lived experience of CBTp	9 (4 Male)	26 (21–65)	8 White British 1 Black British
Lawlor et al. (2015)	Thematic analysis	Purposive	Semi‐structured interviews	Adults with lived experience of psychosis and therapists^a^	8 (8 Male)	35.9 (31–41)	8 White British
Li et al. (2017)	Thematic analysis	Purposive	Semi‐structured interviews	Adults with an ICD‐10 schizophrenia spectrum diagnosis and carers^a^	15 (8 Male)	22 (16–34)	Unknown
Loizou et al. (2024)	Thematic analysis	Convenience	Semi‐structured interviews	Adults who hear voices and therapists	15 (4 Male)	45.3 (26–66)	14 White British
McGowan et al. (2005)	Grounded theory	Purposive	Semi‐structured interviews	Adults with lived experience of CBTp and therapists	8 (4 Male)	37.5 (26–44)	Unknown
Messari and Hallam (2003)	Discourse analysis	Convenience	Semi‐structured interviews	Adults with lived experience of psychosis	5 (4 Male)	40.6 (28–49)	3 White British or Other 2 Black African or Caribbean
Mitchison et al. (2015)	Thematic analysis	Purposive	Semi‐structured interviews	Adults with lived experience of psychosis	44 (24 Male)	26.3 Range unknown	Unknown
Morrison et al. (2021)	Thematic analysis	Purposive	Semi‐structured interviews	Adults experiencing first episode psychosis and their carers	13 (3 Male)	17 (15–19)	10 White British or Other 2 Black African 1 South Asian
Naeem et al. (2016)	Content and question analysis	Purposive	Semi‐structured interviews	Adults with an ICD‐10 schizophrenia spectrum diagnosis, practitioners and carers	33 (18 Male)	32 (18–52)	Unknown
Pain et al. (2008)	Content analysis	Convenience	Semi‐structured interviews	Adults experiencing psychosis and therapists	13 (8 Male)	34.5 (21–64)	Unknown
Pipkin, Armitage, et al. (2021)	Interpretive Phenomenological Analysis	Convenience	Semi‐structured interviews	Adults open to an Early Intervention Psychosis Team with experience of CBTp	8 (2 Male)	34.9 (25–48)	7 White British 1 South Asian
Rathod et al. (2010)	Content analysis	Purposive	Semi‐structured interviews	Adults with a diagnosis of schizophrenia, schizoaffective or delusional disorder and therapists	15 (9 Male)	Unknown	12 Black African or Caribbean 3 South Asian
Slater and Painter (2016)	Participatory Action Research	Purposive	Group game design	Secure inpatients with schizophrenia and therapists	10 (10 Male)	41 (32–50)	9 White British 1 Unknown
Spencer et al. (2023)	Thematic analysis	Purposive	Semi‐structured interviews	Adults with lived experience of CBTp for first‐episode psychosis	10 (5 Male)	28.3 (16–41)	10 White British

^a^ Only data from service users were extracted.

### 3.2 Quality Assessment

Most studies (*k =* 15) were considered high‐quality, describing and justifying, in sufficient detail, decisions made regarding research aims, recruitment, data collection and analysis. The remainder were rated moderate (*k =* 9). Most papers (*k =* 12) included little‐to‐no detail regarding the influence of authors' positionality on the interpretation of results. This, alongside insufficient detail on participant selection or consideration of ethical issues, characterised moderate‐quality studies. Since these issues were not thought to compromise the integrity of the findings of included studies and none were rated low quality, no papers were excluded from the synthesis (Long et al. 2020).

### 3.3 Synthesis

Three themes that capture people's experiences of CBTp were developed: *active collaboration empowers me*; *promoting safety makes me feel more trusting*; *developing shared understandings helps me accept or challenge upsetting experiences*. Relationships between themes are illustrated in Figure 2. They show that CBTp techniques promote acceptance and new meanings if sensitively and collaboratively used and collaboration in CBTp facilitates therapeutic outcomes via empowerment. Both relationships occur only when participants feel safe. Descriptive themes on which analytic themes are based and examples of quotes and codes from which descriptive themes were derived, are presented in the Supporting Information.

**FIGURE 2:**
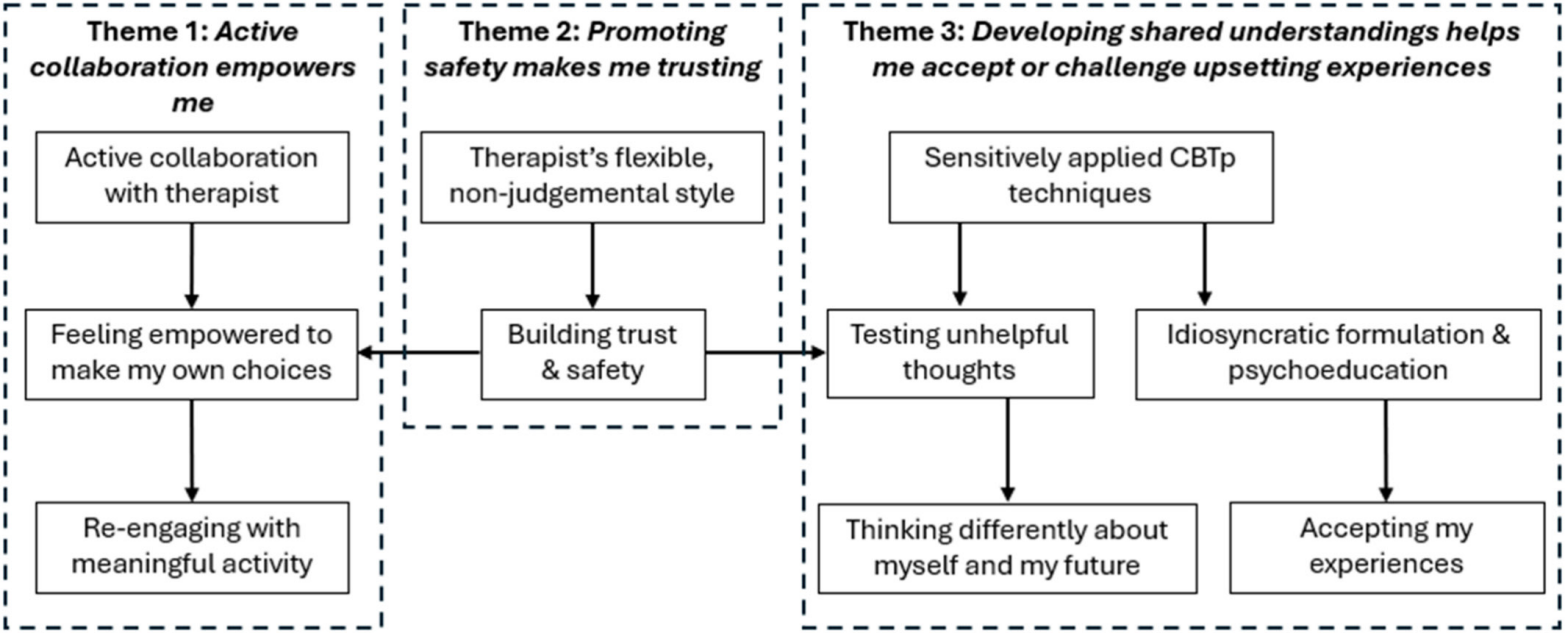
Thematic map illustrating relationships between themes.

### 3.4 Theme 1: Active Collaboration Empowers Me

Participants found CBTp collaborative—they felt they worked alongside their therapist rather than passively receiving therapy. CBTp felt collaborative when therapists avoided jargonistic language, invited participants to negotiate agendas, agree on shared goals and draw out formulations (Aljuhani et al. 2024; H. Dunn et al. 2002; Gibbs et al. 2020; Kilbride et al. 2013; Morrison et al. 2021; Slater and Painter 2016; Spencer et al. 2023). When participants could not recall salient events between sessions, they struggled with collaborative endeavours such as agenda‐setting (Aljuhani et al. 2024; Austin et al. 2021; Dunn et al. 2002; Kilbride et al. 2013). Participants valued reminders of salient events worth discussing in session (e.g., mobile diary; inviting family members into therapy) to compensate for memory difficulties and facilitate collaboration (Aljuhani et al. 2024; Austin et al. 2021).

Participants also found active engagement from therapists in change strategies collaborative. They appreciated when therapists escorted them to in vivo behavioural experiments or completed their own between‐session tasks (Birchwood et al. 2018; Morrison et al. 2021; Pipkin, Armitage, et al. 2021). For this reason, participants appreciated the opportunity to visit distressing locations in virtual reality, as therapists were always alongside them (Holgersen et al. 2023). Collaboration made participants feel equal to their therapist, as a participant in a study on experiences of self‐disclosure in CBTp explained.

*[Therapist] would come to [activity group] with me and she'd like join in with the group. As like a friend not like a therapist as well, you know, so you didn't feel any, you know, it felt, I don't know how to put that into words, but you sort of the same*. (Participant in (Pipkin, Armitage, et al. 2021))

Collaboration rebalanced power by ‘inviting in’ participants to the therapist's position and by therapists ‘moving to’ the participant's position. Participants found this repositioning empowering because they shifted from ‘service user’ to human being. As a participant in a study on people's experiences of CBTp explained.

I'm not just a service user, I'm someone on her level you know, really as a service user you get looked down on and you don't get considered at all, your feelings don't get considered at all when you're ill. (Participant in (Kilbride et al. 2013))

Empowerment through collaboration fostered a sense of agency, enabling participants to establish independence. A participant in a study exploring experiences of formulation in CBTp reflected on the impact of developing formulations collaboratively: ‘I started trying to actually sort of help myself, instead of trying to get other people to help me (…) and when I did, it worked!’ (Participant in (Spencer et al. 2023)). When empowered, participants regained control over their lives, (re)engaging with activities they found meaningful, including occupational (e.g., returning to work or education), social (e.g., reconnecting with family and friends) and prosocial (e.g., helping others) activity (Birchwood et al. 2018; Dellazizzo et al. 2020; Hall et al. 2023; Loizou et al. 2024; Naeem et al. 2016; Pipkin, Armitage, et al. 2021; Rathod et al. 2010).

### 3.5 Theme 2: Promoting Safety Makes me Feel More Trusting

Participants could trust their therapist when they felt safe. Participants felt safe when CBTp therapists were flexible and non‐judgemental (Aljuhani et al. 2024; Birchwood et al. 2018; Dunn et al. 2002; Hall et al. 2023; Kilbride et al. 2013; Morrison et al. 2021; Pipkin, Armitage, et al. 2021). Flexibility in the location of therapy made participants feel physically safe: ‘we started going [to have the therapy at] my Grans and it was alright … Because I feel safe at my Grans’ (Participant in (Birchwood et al. 2018)). When therapists had access to mobile mood diaries, they could anticipate distress and plan sessions flexibly, which participants felt fostered psychological safety: “it did sort of make me feel safe that I knew he was keeping an eye on those graphs … that was really nice” (Participant in (Austin et al. 2021)). Psychological safety was also felt by participants when therapists were non‐judgemental, as they could share their experiences openly, without fear of criticism: “I think that just kind of helps build that relationship that you feel you can be super honest with them because like there's no judgement from them at all*”* (Participant in (Pipkin, Armitage, et al. 2021)). Some participants felt psychologically safer when their therapist's demographics met their expectations (Gibbs et al. 2020; Aljuhani et al. 2024; Rathod et al. 2010). For instance, when cultural beliefs normalised gender segregation, participants (especially non‐White participants) felt they could share more openly with therapists of the same gender (Rathod et al. 2010; Aljuhani et al. 2024).

Safety was also established by sensitive introduction of change strategies, which participants only found helpful when they felt psychologically flexible (Kilbride et al. 2013; Mitchison et al. 2015; Morrison et al. 2021; Spencer et al. 2023). As one participant described in a study on experiences of CBTp ‘You've got to be ready and motivated for it cos […] you need to be fairly open‐minded.’ (Participant in (Kilbride et al. 2013)). For another participant in a study on experiences of CBTp homework ‘the last few times I declined because it was to try and improve what I was thinking and the thoughts and voices weren't true’ (Participant in (Dunn et al. 2002)). Where participants were not ready to engage meaningfully with cognitive restructuring, examining appraisals communicated an implicit and unhelpful denial of their experiences, compromising their safety.

Conversely, trust was difficult to establish for participants when talking openly felt intimidating, for instance, worrying that talking increases distress or that information would be shared, risking judgement from others (Aljuhani et al. 2024; Kilbride et al. 2013; Mitchison et al. 2015; Morrison et al. 2021; Pipkin, Armitage, et al. 2021). For example, a participant in a study on young inpatients' experiences of CBTp ‘disclosed ambivalence over telling psychologists things … Stated that he was worried they would “spill secrets”,’ (Mitchison et al. 2015). Talking felt intimidating for participants with historic experiences of the mental health system as oppressive (McGowan et al. 2005; Messari and Hallam 2003; Mitchison et al. 2015; Pipkin, Armitage, et al. 2021). The authors of the study exploring self‐disclosure in CBTp explained the impact of prior experiences on disclosure: ‘Three participants additionally feared physical consequences; opening up about their psychosis would result in them being “locked up” and force‐fed medication’ (Pipkin, Armitage, et al. 2021). Participants attributed oppressive experiences to professionals' inflexibility to develop a shared understanding, leading to mistrust of mental health staff that, to some, included CBTp therapists ‘no, I just suffer, I won't ask [mental health service] for help’ (Participant in (Hall et al. 2023)).

Once participants trusted their therapists, they could trust other people (Birchwood et al. 2018; Health Quality Ontario 2018; Kilbride et al. 2013; Lawlor et al. 2015; Mitchison et al. 2015; Morrison et al. 2021; Pipkin, Armitage, et al. 2021). For instance, a participant in a study of paranoia in the therapeutic relationship shared, *‘*it restored my faith in human beings … I'm a little less guarded around people now … if you can trust one person maybe you can extend that trust to other people.’ (Participant in (Lawlor et al. 2015)). The ability to develop a trusting relationship helped this participant feel safer in (re)connecting with others elsewhere. Likewise, when participants trusted their therapist, they engaged better with change strategies (Birchwood et al. 2018; Holgersen et al. 2023; Slater and Painter 2016). For example, a CBTp‐based board game developed by forensic inpatients exemplified the mobilising effect of trust, whereby trust moved players forward by ‘giving things a go despite being afraid and the effects of particular interventions’ (Slater and Painter 2016) and mistrust moved players backwards.

### 3.6 Theme 3: Developing Shared Understandings Helps me Accept or Challenge Upsetting Experiences

Participants found psychoeducation normalising. A shared understanding of, for instance, the prevalence of unshared experiences made participants feel less alienated (Aljuhani et al. 2024; Birchwood et al. 2018; Hall et al. 2023; Kelly et al. 2012; Kilbride et al. 2013; Messari and Hallam 2003; Morrison et al. 2021; Pipkin, Armitage, et al. 2021; Spencer et al. 2023). For example, in a study on the acceptability of CBT for commanding voices, one participant explained psychoeducation ‘was helpful; let me feel that I wasn't alone. I felt a little bit better in myself’ (Participant in (Birchwood et al. 2018)).

Participants found formulation helpful because it improved their understanding of the maintenance and/or development of upsetting experiences (Birchwood et al. 2018; Dellazizzo et al. 2020; H. Dunn et al. 2002; Gibbs et al. 2020; Hall et al. 2023; Health Quality Ontario 2018; Kilbride et al. 2013; Loizou et al. 2024; Messari and Hallam 2003; Morrison et al. 2021; Pain et al. 2008; Pipkin, Armitage, et al. 2021; Spencer et al. 2023). As one participant in a study on experiences of formulation in CBTp explained how shared understanding through formulation destigmatised experiences.

Interviewer: You said that it helped, seeing it [written] down on paper?

Participant: Knowing that I wasn't crazy.

Interviewer: And how did writing it on paper help you to see that you were not crazy?

Participant: Like, it all came together … why I'm hearing the voices. (Participant in (Spencer et al. 2023))

However, when insufficiently detailed, participants found formulations irrelevant and when overly detailed, formulations were overwhelming (Dunn et al. 2002; Gibbs et al. 2020; Pain et al. 2008; Spencer et al. 2023). Therefore, participants only found the formulation helpful when it was accessible. Accessible formulation created a shared understanding of the idiosyncratic origins of unshared experiences, providing participants a sense of clarity that demystified them, thereby reducing distress (Dunn et al. 2002; Messari and Hallam 2003; Naeem et al. 2016; Rathod et al. 2010; Spencer et al. 2023).

Decreasing alienation via psychoeducation and facilitating understanding via formulation repositioned distressing experiences from aversive to acceptable. For example, a participant in a study on participants' priorities in CBTp outcomes ‘I've accepted the fact that I do hear those voices and not sort of like I used to, pretend that it wasn't happening, because I was too scared to admit to it’ (Participant in (Loizou et al. 2024)). Acceptance allowed participants to lead meaningful lives alongside unshared experiences, making them less impactful and, in turn, upsetting (Birchwood et al. 2018; Dellazizzo et al. 2020; Hall et al. 2023; Kilbride et al. 2013; Loizou et al. 2024; Messari and Hallam 2003; Pipkin, Armitage, et al. 2021; Slater and Painter 2016). For instance, in a grounded theory study on change mechanisms in CBTp, one participant explained therapy ‘makes me feel like I haven't got a mental health problem, I can just get on with my life. I can get on with myself more’ (Participant in (Hall et al. 2023)).

Thus, when a shared understanding involved normalising distressing experiences, participants accepted them. In contrast, when a shared understanding identified unhelpful appraisals as maintaining distress, participants appreciated the opportunity to challenge them. Participants valued techniques like evidential analysis and behavioural experiments in this respect because they could use them to question unhelpful appraisals and in turn reduce distress in daily life (Dunn et al. 2002; Hall et al. 2023; Health Quality Ontario 2018; Kilbride et al. 2013; Messari and Hallam 2003; Pain et al. 2008; Slater and Painter 2016; Spencer et al. 2023).

Participants found that testing unhelpful thoughts helped overcome cognitive biases identified by their shared understanding, demonstrated by a participant's experience in a mixed‐methods audit of the implementation of CBTp in Canada: ‘the whole idea with CBT is to get cognitive restructuring, where you start to think in terms of, logically; you start to think logically and your anxiety and all that goes down.*’* (Participant in (Health Quality Ontario 2018)). Identifying biases and thinking in a more balanced way directly reduced distress (Dunn et al. 2002; Health Quality Ontario 2018; Loizou et al. 2024; McGowan et al. 2005). Testing unhelpful appraisals of upsetting experiences also reduced distress, like finding voices less scary or powerful (Birchwood et al. 2018; Hall et al. 2023; Kilbride et al. 2013). Participants found they could control experiences like voices when they viewed them in less distressing ways (Birchwood et al. 2018; Dellazizzo et al. 2020; H. Dunn et al. 2002; Hall et al. 2023; Loizou et al. 2024; Morrison et al. 2021; Rathod et al. 2010; Slater and Painter 2016; Spencer et al. 2023). As a participant in a clinical trial of CBTp with or without antipsychotics reported ‘Although I still have like the symptoms and stuff and I still get it every now and then pretty bad, I have like more like control over it.’ (Participant in (Morrison et al. 2021)).

Like acceptance, controlling experiences made them less impactful, leading participants to think more positively about themselves, the world and their future because they could move on with their lives (Hall et al. 2023; Kilbride et al. 2013; Messari and Hallam 2003; Morrison et al. 2021; Spencer et al. 2023). A participant in a study of cultural adaptations in CBTp described the ubiquitous impact of thinking differently ‘It was helpful. It helped me with thinking clearly. I started going to work. It helped me to express my emotions. It helped me with catharsis. Now I don't argue or fight with my family members.’ (Participant in (Naeem et al. 2016)).

## 4 Discussion

Following its global implementation in mental health services, this review meta‐synthesised qualitative literature exploring clients' experiences of CBTp, with the aim of identifying routes to improve its efficacy. We developed three interconnected analytic themes. Participants found active collaboration empowering to the extent that some re‐engaged with meaningful activity and developing shared understandings with therapists helped participants accept or challenge upsetting experiences. Centrally, though, participants found these processes depended on trust and safety, fostered by therapeutic relationships.

When therapists worked collaboratively, participants felt an equalisation of power in the relationship. Prior meta‐syntheses of both therapist and participant experiences of CBTp reported that collaboration redistributes power in this way (Wood et al. 2015). “Radical collaboration” is considered essential to promoting engagement with CBTp (Chadwick 2008). Yet, our findings indicate that collaboration can directly facilitate therapeutic gains. Consistently, a recent meta‐ethnographic review of qualitative studies exploring therapeutic relationships in CBTp found that collaboration fosters safety and trust that facilitate change as they become internalised (Pipkin, Hogg, and Armitage 2021). Our findings suggest that safety and trust facilitate change because collaboration empowers participants. As hypothesised by social rank theory, participants may feel empowered by collaboration as it may implicitly invite participants to consider themselves equal to, rather than dominated by, others (Birchwood et al. 2000).

Participants found that developing shared understandings in CBTp changed their appraisals of their experiences. Medical models of psychopathology often define recovery as the ‘remission’ of distressing experiences, if not individual conformity to a medical understanding of their experiences (e.g., as lifelong ‘symptoms’; Oorschot et al. 2012). Yet, in line with prior meta‐syntheses, our model demonstrates that *imposed* understandings were harmful, whereas *shared* understandings facilitated therapeutic gains (Berry and Hayward 2011; Cox and Miller 2021; Wood et al. 2015). Readers may find our use of the phrase ‘*challenging* experiences’ in this Theme surprising since it may incur a sense of imposing understanding. From our perspective, alternative phrasing (e.g., ‘*evaluate* experiences’) communicates a position that experiences are not truly real and/or distress not valid and thus subject to evaluation. We use ‘challenge’ with the aim of honouring distress experienced by participants that therapists face, together, thereby developing a shared understanding.

Our findings suggest the development of shared understanding reduces distress in itself, either directly (e.g., challenging distressing beliefs) or indirectly (e.g., accepting distressing voices). It is not merely the mutuality of understanding, but the collaborative construction of meaning that appears significant. Variation in the subjective changes to cognition in our model, including thinking differently about oneself, the future and experiences, may indicate that CBTp changes cognitive *style* (i.e., increased psychological flexibility) alongside *content* (i.e., unhelpful appraisals). Adopting alternative perspectives across multiple domains may indicate greater flexibility. Indeed, psychological inflexibility is associated with increased distress among people with psychosis (Pittman et al. 2024).

A meta‐ethnographic review recently concluded that safe therapeutic relationships facilitate therapeutic gains via the internalisation of attachment security (Pipkin, Hogg, and Armitage 2021). Insofar as trusting therapists empowered participants to trust others, we replicate these findings. Our model extends the work of Pipkin and colleagues by implying the promotion of safety and trust is pivotal in facilitating gains from CBTp techniques. That safe relationships are not in isolation therapeutic may explain why assessment and engagement alone are not associated with therapeutic gains (Dunn et al. 2012). Safety acting as a condition of engagement is likely secondary to threatening, unsafe experiences that are overwhelmingly common in people with psychosis, rather than distressing beliefs/sensations (Hardy et al. 2022). That is, CBTp engagement may be complicated not only by suspiciousness or malevolent voices, but also by trauma‐related beliefs and hypervigilance. Whilst gold‐standard CBTp is considered trauma‐informed at minimum (Brabban et al. 2017), trauma and its impact are noticeably missing from our findings. Disclosures of this kind are not typically offered spontaneously and as such require explicit exploration in future qualitative studies (Read 2007).

### 4.1 Strengths and Limitations

To our knowledge, the pooled sample of the present meta‐synthesis is among the largest to date. Such a large sample allowed for diverse individual experiences of therapy. Our findings reflect both positive and negative experiences of various CBTp components that nuanced our themes. For instance, formulation is helpful when idiosyncratic and CBTp techniques helpful when sensitively applied. Further, the exclusive focus on service user perspectives identified the importance of promoting safety and trust in CBTp hitherto acknowledged, but not centralised, in prior meta‐syntheses (Wood et al. 2015; Berry and Hayward 2011). Our sample size therefore represents an important strength of our review.

However, the included studies were predominantly published in Western countries and, where reported, samples were largely White, so our findings may only reflect the experiences of White, Western people. Whilst at least two studies on the experiences of CBTp among minoritised ethnic groups were included in this review, neither exclusively reported service user perspectives (Naeem et al. 2016; Rathod et al. 2010). As such, their findings did not substantially influence our results. Because some principles of CBTp may counter non‐Western cultural philosophies (e.g., empiricism over intuition), non‐White people may have different, even harmful, experiences of the approach (Isiwele et al. 2025). As above, these experiences may be missing from our themes.

To capture a holistic perspective on contemporary CBTp practice, studies involving augmented CBTp were included. CBTp was supplemented by several additions, such as virtual reality or smartphone apps, introducing heterogeneity in the intervention of interest and participant experiences thereof. Whether the experience of innovative CBTp differs substantively from ‘standard’ practice (and thus subject to separate analytic themes) is therefore unclear from our findings. Excluding data from therapists, family members/carers and laypeople may also have limited our results. For example, people with whom participants have close personal relationships may notice therapeutic outcomes that are not apparent to participants themselves (Health Quality Ontario 2018). Likewise, training and therapy manuals may enable therapists to better identify the utility of specific techniques than participants (Berry and Hayward 2011). As such, including this data may have led to the development of additional descriptive themes that further nuanced our analytic themes.

### 4.2 Clinical and Research Implications

Health and care provision is globally shifting away from a ‘one‐size‐fits‐all’ approach towards precision medicine (i.e., targeted interventions for the right individual at the right time; WHO 2026). For some, the application of precision medicine to psychological therapies, including those for psychosis, implies a menu of short‐term, mechanism‐focused interventions (Pillny et al. 2026). Our findings suggest that precision medicine may translate to the *way* therapies are delivered, as opposed to increasingly narrow intervention targets. That is, based on our findings, precision medicine in CBTp should be aimed at fostering idiosyncratic, psychologically safe and collaborative practices. Whilst the importance of the therapeutic relationship in psychological interventions is established, our findings demonstrate the extent of this importance that should be carefully considered as psychological interventions for psychosis become increasingly mechanism‐focused (Brabban et al. 2017; Pillny et al. 2026).

Likewise, global economic pressures are making scalable talking therapies, deliverable by non‐psychological professionals or AI chatbots, more attractive (WHO 2024; Zhang et al. 2025). Non‐specialists and chatbots may require strict adherence to manualised protocols to maintain model fidelity, but our findings indicate that CBTp engagement hinges on interpersonal safety, collaboration and sensitivity. As scalability grows, therapist meta‐competencies facilitating these factors should therefore be considered essential in CBTp training alongside technical competencies (Roth and Pilling 2013).

Our overlap with findings of prior meta‐syntheses suggests CBTp has remained acceptable over time (Cox and Miller 2021; Pipkin, Hogg, and Armitage 2021; Wood et al. 2015). Since acceptability informs recommendations in national clinical guidelines, our findings support the ongoing provision of CBTp in mental health services (NICE 2025). The subjective therapeutic gains we identified broadly map onto improvements in daily functioning and quality of life, consistent with an umbrella review of meta‐analyses of CBTp (Solmi et al. 2023). Indeed, a content analysis of CBTp problem lists suggested over half of service users' priority problems were not psychosis‐specific, with one fifth of participants not listing any psychosis‐related problems (Morrison et al. 2023). In future evaluation of CBTp efficacy, our findings support the use of primary outcomes other than symptom change, such as quality of life or subjective recovery, in line with the priorities of people with lived experience of psychosis (Byrne and Morrison 2014).

## 5 Conclusion

In the largest meta‐synthesis to date that centred service user experiences, we demonstrated that CBTp techniques reduce distress if sensitively and collaboratively used and only when participants feel safe. We extended prior meta‐syntheses of CBTp by directly linking common therapeutic factors to therapeutic outcomes, via empowerment. In the advent of precision medicine, therapist meta‐competencies aimed at promoting collaboration, safety and idiosyncrasy should be prioritised in the future of CBTp practice and may offer a promising avenue to improve its efficacy. Though, future research should integrate more diverse perspectives to further nuance the understanding of the experience of CBTp.

## Funding

This research received no specific grant from any funding agency, commercial or not‐for‐profit sectors. K.B. is supported by the National Institute for Health Research (NIHR) Manchester Biomedical Research Centre (NIHR 203308).

## Conflicts of Interest

The authors declare no conflicts of interest.

## Supporting information


**Supplementary Table 1.** Examples of key quotes and example codes that contributed to descriptive themes and subthemes.
**Supplementary Table 2.** Matrix demonstrating descriptive theme endorsement by study.

## Data Availability

The data that support the findings of this study are available from the corresponding author upon reasonable request.
