# Supplementary material for: An Updated Systematic Review and Meta‐Synthesis of Service User's Experiences of Cognitive Behavioural Therapy for Psychosis (CBTp)
Source: Clin Psychol Psychother. 2026 Nov 5;33(6):e70356. doi: 10.1002/cpp.70356 (PMC13740465; doi:10.1002/cpp.70356)
Supplement: Supplementary file 1 [file CPP-33-e70356-s001.docx]

| **Key quotes** | **Example code(s)** | **Descriptive theme and subthemes** |
| --- | --- | --- |
| *“patients … sought validation from therapists through demonstration that they were taking a genuine stance of treating patients as people with equal status rather than just as “mental” patients.”* (Rathod et al., 2010)  *“anything I did say I felt you know that no-one’s gonna judge me.”* (Kilbride et al., 2013) | Collaboration; therapist doing things with me; Location; Feeling understood; Being listened to; Talking about experiences; My own words; Idiosyncrasy | **Therapeutic style**   - Collaborative - Non-judgemental - Treating people with dignity - Person-centred |
| *“CBT can be used with delusions because you can do thought records to challenge the delusions. It’s really a wonderful thing.”* (HTO, 2018)  *‘it would give me proof .. arguments for and against’* (Dunn et al., 2002) | Understanding maintenance; Problem lists; Structure is restrictive; Psychoeducation is good; Normalising helps; Putting things into practice; Weighing up evidence | **CBT techniques**   - Formulation & structure - Shared goals - Psychoeducation & normalising - Experiments & practice |
| *“I can’t always remember, well, so how was it I was feeling yesterday”* (Austin et al., 2021)  *“they tell me, the medical establishment, tell me are hallucinations, I still believe they are real”* (Messari & Hallam, 2003) | Not enough information; Homework is onerous; I’m not ill; Formulation is upsetting; Therapy is draining; Worries about technology; Technology helps me remember | **Non-specific factors**   - Accessibility - Unshared reality - Distress - Integrating technology |
| *“I think I was just braver […] I wasn’t that weak person that closed down.”* (Hall et al., 2023)  *“it restored my faith in human beings… I’m a little less guarded around people now… if you can trust one person maybe you can extend that trust to other people.*” (Lawlor et al., 2015) | Coping better; thinking rationally; feeling more confident; Weight lifted; (Re)starting hobbies; Enjoying quality time; Agency; Independence; Gaining control over voices; No effect on mood | **Outcomes**   - Coping skills - Thinking differently - Taking back control - No benefit |

**Supplementary Table 1.** Examples of key quotes and example codes that contributed to descriptive themes and subthemes.

**Supplementary Table 2.** Matrix demonstrating descriptive theme endorsement by study

| **Study** | **Descriptive theme** | | | |
| --- | --- | --- | --- | --- |
|  | **Therapeutic style** | **CBT techniques** | **Non-specific factors** | **Outcomes** |
| Aljuhani et al. (2024) | X | X | X | - |
| Austin et al. (2021) | X | - | X | - |
| Birchwood et al. (2018) | X | X | X | X |
| Dellazizzo et al. (2020) | - | X | - | X |
| Dunn et al. (2002) | X | X | X | X |
| Gibbs et al. (2020) | X | X | X | X |
| Hall et al. (2023) | X | X | - | X |
| Holgersen et al. (2023) | X | X | X | - |
| Health Technology Ontario (2018) | X | X | - | X |
| Kelly et al. (2012) | - | X | - | - |
| Kilbride et al. (2013) | X | X | X | X |
| Lawlor et al. (2015) | X | - | - | X |
| Li et al. (2017) | - | - | X | - |
| Loizou et al. (2024) | - | X | - | X |
| McGowan et al. (2005) | X | X | X | X |
| Messari & Hallam (2003) | - | X | X | X |
| Mitchison et al. (2015) | - | X | X | X |
| Morrison et al. (2021) | X | X | - | X |
| Naeem et al. (2016) | - | X | X | X |
| Pain et al. (2008) | - | X | X | X |
| Pipkin et al. (2021) | X | X | - | X |
| Rathod et al. (2010) | - | X | X | X |
| Slater & Painter (2016) | X | X | - | X |
| Spencer et al. (2023) | X | X | X | X |
